# Psoriasis immunometabolism: progress on metabolic biomarkers and targeted therapy

**DOI:** 10.3389/fmolb.2023.1201912

**Published:** 2023-06-19

**Authors:** Evangelia Sarandi, Sabine Krueger-Krasagakis, Dimitris Tsoukalas, Polytimi Sidiropoulou, George Evangelou, Maria Sifaki, Gottfried Rudofsky, Nikolaos Drakoulis, Aristidis Tsatsakis

**Affiliations:** ^1^ Laboratory of Toxicology and Forensic Sciences, Medical School, University of Crete, Heraklion, Greece; ^2^ Metabolomic Medicine, Health Clinics for Autoimmune and Chronic Diseases, Athens, Greece; ^3^ Dermatology Department, University Hospital of Heraklion, Heraklion, Greece; ^4^ European Institute of Molecular Medicine, Rome, Italy; ^5^ 1st Department of Dermatology-Venereology, Faculty of Medicine, “A. Sygros” Hospital, National and Kapodistrian University of Athens, Athens, Greece; ^6^ Research Group of Clinical Pharmacology and Pharmacogenomics, Faculty of Pharmacy, School of Health Sciences, National and Kapodistrian University of Athens, Athens, Greece; ^7^ Clinic of Endocrinology and Metabolic Disorders, Cantonal Hospital Olten, Olten, Switzerland

**Keywords:** psoriasis, T cell, keratinocyte, glycolysis, lipid metabolism, TCA, biomarkers, metabolic targets

## Abstract

Psoriasis is a common inflammatory disease that affects mainly the skin. However, the moderate to severe forms have been associated with several comorbidities, such as psoriatic arthritis, Crohn’s disease, metabolic syndrome and cardiovascular disease. Keratinocytes and T helper cells are the dominant cell types involved in psoriasis development via a complex crosstalk between epithelial cells, peripheral immune cells and immune cells residing in the skin. Immunometabolism has emerged as a potent mechanism elucidating the aetiopathogenesis of psoriasis, offering novel specific targets to diagnose and treat psoriasis early. The present article discusses the metabolic reprogramming of activated T cells, tissue-resident memory T cells and keratinocytes in psoriatic skin, presenting associated metabolic biomarkers and therapeutic targets. In psoriatic phenotype, keratinocytes and activated T cells are glycolysis dependent and are characterized by disruptions in the TCA cycle, the amino acid metabolism and the fatty acid metabolism. Upregulation of the mammalian target of rapamycin (mTOR) results in hyperproliferation and cytokine secretion by immune cells and keratinocytes. Metabolic reprogramming through the inhibition of affected metabolic pathways and the dietary restoration of metabolic imbalances may thus present a potent therapeutic opportunity to achieve long-term management of psoriasis and improved quality of life with minimum adverse effects.

## 1 Introduction

Plaque-type psoriasis is a common immune-mediated inflammatory skin disease involving skin-homing pathogenic T cells, dendritic cells, keratinocytes and their cytokines in its complex pathogenesis. An imbalance between Th1/Th17 axes seems to be a common feature in the psoriatic cascade through the secretion of pro-inflammatory cytokines that promote keratinocyte proliferation (Vollmer et al., 1994; Lowes et al., 2008; Hu et al., 2021). In synergy with Th1 and Th17, the IL-22-secreting T cells have been identified as indispensable contributors to the development of psoriatic lesions along with IL-23-producing myeloid cells (Schön and Erpenbeck, 2018). In addition, psoriatic lesions are characterized by impaired regulation of excess immune response due to functional defects of regulatory T cells (Tregs) (Sugiyama et al., 2005). Keratinocytes are appreciated as initiators of the immune response through the production of autoantigens and in the establishment of the disease through the crosstalk with adaptive immunity cells that aggravates the cytokine milieu and intensifies chronic inflammation (Lande et al., 2007; Hawkes et al., 2017; Zhou et al., 2022).

Epidemiological data on psoriasis are scarce, considering that only 19% of the countries worldwide have available data. However, it is estimated that psoriasis prevalence ranges from 0,11% in East Asia to 3,61% in Denmark and 3% in the United States, collectively affecting over 125 million people worldwide (Parisi et al., 2020; Armstrong et al., 2021; National Psoriasis Foundation, 2022). Psoriasis is characterized by increased comorbidities, such as psoriatic arthritis, Crohn’s disease and cardiometabolic diseases (Takeshita et al., 2017) and significant quality of life compromise (Stern et al., 2004). Several studies have indicated that metabolic complications implicate a higher risk of developing psoriasis and directly correlate with disease severity and worse therapeutic outcomes (Polic et al., 2018; Hao et al., 2021; Lee et al., 2021). Since different subsets of helper cells have been considered key players in psoriasis’ immunological pathways, interest has increasingly been focused on elucidating the impact of metabolism on the immune system’s function of psoriasis patients.

In this sense, the evolving field of immunometabolism, linking immunology and metabolism, could provide critical points for investigating how metabolic cellular reactions and processes may control immunity and inflammation, offering novel tools for monitoring and managing immune-mediated diseases, including psoriasis.

Given that emerging evidence suggests targeting specific metabolic events as a strategy to limit cutaneous inflammation, this review will first focus on the potential mechanisms underlying the association between the metabolic reprogramming of the critical cell players and inflammatory skin responses of psoriasis. Potential metabolic biomarkers and therapeutic anti-psoriatic approaches integrating immune/metabolic responses from the published literature are also summarized.

## 2 Pathogenetic mechanism of psoriasis

Psoriasis is widely regarded as a multifactorial immune-related disease triggered by environmental factors in the background of genetic predisposition. Although the precise pathogenetic mechanisms have yet to be deciphered, psoriasis seems to be controlled by endogenous and exogenous factors.

The genetic basis of psoriasis has been supported by family and twin studies suggesting a higher incidence of psoriasis within families and in monozygotic twins than in dizygotic twins. Genome-wide association studies have indicated thirteen different genetic regions, known as Psoriasis Susceptibility (PSORS)1-13, contributing to disease susceptibility. The PSORS1, located within the Major Histocompatibility Complex (MHC) on chromosome 6p21, is the primary locus for psoriasis susceptibility. The HLA-Cw6 is one of this region’s most strongly associated alleles affecting disease onset, course, severity, comorbidities, and treatment outcomes (Chen and Tsai, 2018). Although the exact role of HLA-C in psoriasis development remains unclear, there seems to be an interplay/relation between HLA-Cw6 status and immune dysregulation in psoriasis patients.

While genetic factors are well documented in psoriasis, several environmental triggers, such as mechanical skin trauma and inflammation, infections, drugs, smoking, excessive alcohol intake, mental stress and microbiota/diet, and epigenetic modifications, may induce, trigger or exacerbate the disease.

The mechanism through which external factors contribute to the irregular proliferation of keratinocytes and the progression to psoriatic lesions involves innate and adaptive immunity cells (Armstrong and Read, 2020). Briefly, after exposure to non-genetic triggers of psoriasis, damaged keratinocytes express antimicrobial peptides (AMPs), including cathelicidin (LL-37), β-defensins, and S100 proteins (psoriasin and Koebnerisin), self- DNA and pro-inflammatory cytokines. The most well-studied AMP is LL-37 which, when produced by damaged keratinocytes, forms aggregates with self-DNA and, along with secreted pro-inflammatory cytokines, results in the activation of plasmacytoid dendritic cells (pDCs) and natural killer cells (NK). Activated pDCs and NK cells secrete interferon-alpha (IFN-α), interferon-gamma (IFN-γ), and tumor necrosis factor-alpha (TNF-α), which activate myeloid dendritic cells (mDCs) to migrate in the lymph node. In turn, mDCs produce interleukin-12 (IL-12) and interleukin-23 (IL-23), resulting in the differentiation of naïve T cells to Th-1 and Th-17 cells, respectively. The differentiation step of naïve T cells into effector cells, mainly Th17 cells, is critical for disease maintenance. Activated T cells migrate from the lymph nodes to the skin microenvironment where Th17 cells secrete interleukin-22 (IL-22), interleukin-17 (IL-17) and TNF-α, and activated Th1 cells secrete TNF-α and IFN-γ. These secreted cytokines induce keratinocyte proliferation, differentiation and further production of antimicrobial peptides and pro-inflammatory cytokines in a feedback loop that causes psoriasis symptomatology. In established psoriasis, premature keratinocytes are abundant throughout the epidermis, causing parakeratosis and the scales along with increased levels of infiltrated immune cells in the epidermis (DCs, macrophages and T cells) and the dermis (mainly neutrophils and some T cells) (Nestle et al., 2009; Armstrong and Read, 2020; Ni and Lai, 2020).

The presence of autoantigens which trigger the expansion of autoreactive T cells has prompted researchers to investigate psoriasis as an autoimmune disease. Up to date, LL-37, ADAMTSL5, PLA2G4D and keratin 17 are the most well-studied autoantigens of psoriasis associated with the presence of autoreactive T cells. On the other hand, antibodies against the autoantigens anti-LL37 and ADAMTSL6 have been observed in the serum of patients with psoriatic arthritis and some patients with psoriasis, supporting the autoimmune nature of psoriasis (Frasca et al., 2018; Yuan et al., 2019; Ten Bergen et al., 2020). However, the lack of adequate data and the specific role of autoantibodies in the inflammatory cascade that initiate psoriasis have stalled the unwinding progression to the disease etiology.

## 3 Metabolic aspects of psoriasis

One of the most common comorbidities of psoriasis is metabolic syndrome (MetS), including related cardiometabolic disturbances such as insulin resistance (IR), obesity, diabetes and dyslipidemia. Increasing evidence suggests that patients with higher psoriasis area and severity index (PASI) score have more severe MetS phenotype indicating potential interconnection between psoriasis and MetS. Studies focusing on the overlapping mechanisms of psoriasis and metabolic diseases have yielded encouraging results for unraveling disease pathogenesis, prompting researchers to further study the metabolic aspects of psoriasis. As described in a recent review, endoplasmic reticulum stress, reactive oxygen species, gut dysbiosis and adipocytokines levels comprise the main factors linking psoriasis pathogenesis with MetS (Hao et al., 2021). However, in a separate study, psoriasis severity was associated with insulin resistance independently of MetS. Insulin resistance is a metabolic disturbance occurring in obese but also lean-healthy individuals as an early indicator of a pre-diabetic state (Polic et al., 2018). This was also observed in psoriasis, where body mass index (BMI) and waist circumference did not affect IR association with disease severity (Eckel et al., 2015). However, patients with psoriasis are not monitored for insulin resistance and other metabolic complications by their physicians, preventing other comorbidities. Metabolic state profiling is a relatively new field in clinical practice due to the limited established biomarkers, yet it fills the gap in prevention, prediction, diagnosis of metabolic comorbidities and treatment response monitoring as part of precision medicine (Newgard, 2017).

### 3.1 Metabolic reprogramming in psoriasis

**FIGURE 1:**
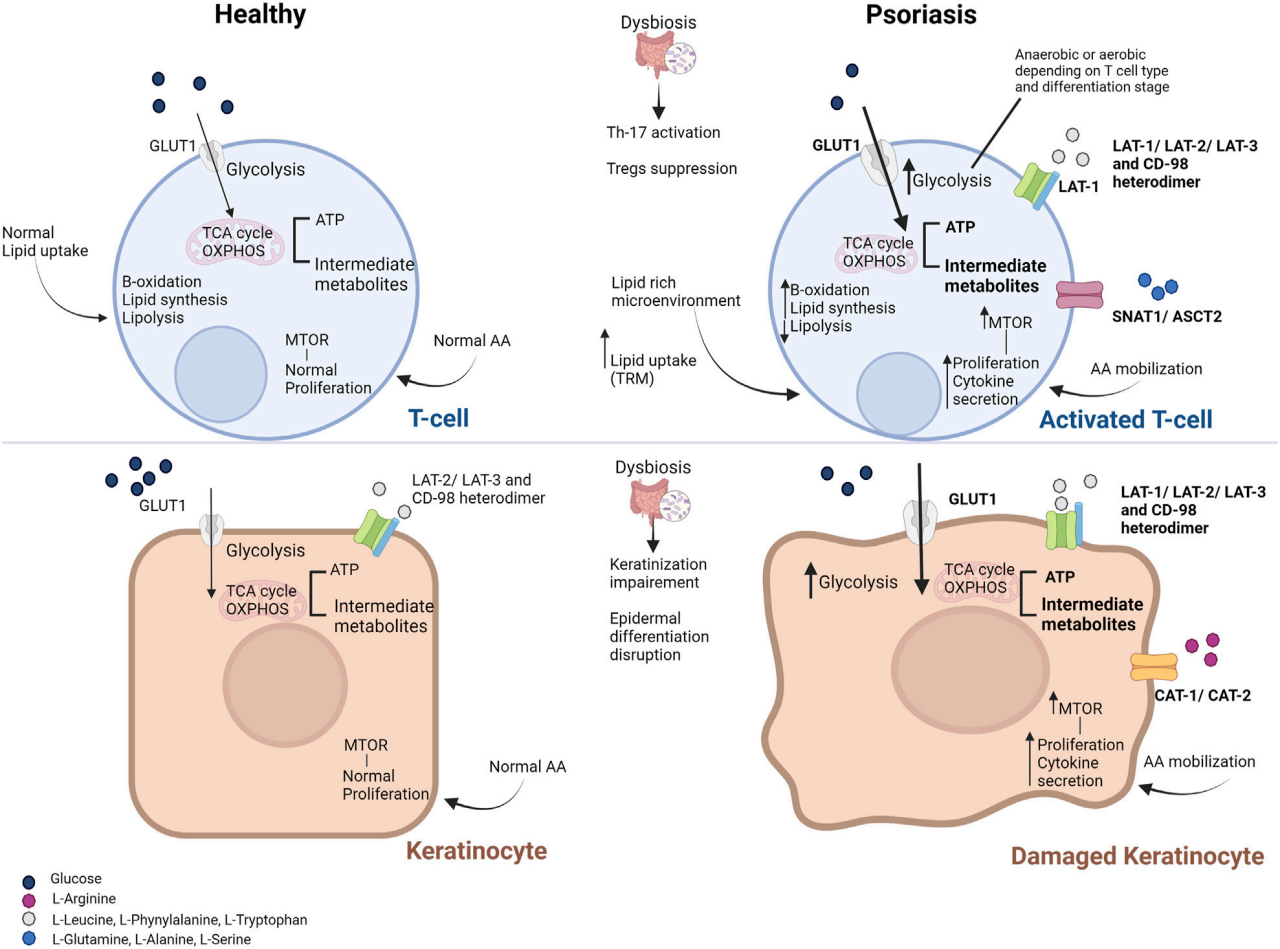
Metabolic derangements in T cells (top) and keratinocytes (bottom) in psoriasis. Metabolic derangements in activated T cells aim to cover their needs in energy and substrates (enhanced glycolysis and downstream energy metabolism pathways, shown in bold). However, T cells residing in the skin (such as memory T cells) rely on the uptake of lipids from the lipid-rich and low-glucose microenvironment. Damaged keratinocytes upregulate glycolytic pathways to cover their proliferative demands as well (shown in bold). Activation of the mammalian target of the rapamycin (mTOR) pathway via the increased amino acid availability and the abundance of pro-inflammatory mediators facilitates the high proliferation and cytokine secretion by activated T cells and keratinocytes. Increased influx of glucose and amino acids is facilitated by the overexpression of the respective transporters on the cell membrane of T cells and keratinocytes (shown in bold). Only primary substrates are shown for each transporter. Gut microbiome deregulation and the aberrant production of microbial metabolites result in intestinal permeability, activation of pro-inflammatory Th-17 cells and suppression of Tregs, while it promotes the disruption of the epidermis integrity. Abbreviations: AA, amino acids; ASCT, neutral amino acid transporter; GLUT1, glucose transporter; LAT, L-type amino acid transporter; mTOR, mammalian target of the rapamycin; OXPHOS, oxidative phosphorylation; SNAT, odium-coupled neutral amino acid transporter; TCA, tricarboxylic acid cycle; Th-17, T-helper 17 cells; Treg, T regulatory cells; TRM, tissue-resident memory cells [Modified from (21)].

Immune cell infiltration and cytokine-driven aberrant keratinocyte overgrowth and differentiation are prominent features of psoriasis lesions. Both keratinocytes and immunocompetent cells can actively metabolize nutrients in their microenvironment (Nestle et al., 2009), suggesting increased cell nutritional requirements in psoriatic skin. As a result, proliferating epidermal and immune cells seem to undergo similar or specific metabolic rewiring to fuel the anabolic pathways and cytokine release (Kamleh et al., 2015) (Figure 1).

#### 3.1.1 Derangements in cellular metabolism

Under normal conditions, keratinocytes use glucose to produce energy via the glycolysis pathway and largely depend on glucose transporter 1 (GLUT1) for glucose uptake. Upon activation, such as in psoriasis-associated hyperplasia, GLUT1 expression is upregulated to meet the proliferating keratinocytes’ elevated metabolic and biosynthetic demands/needs (Cibrian et al., 2020a; Pålsson-McDermott and O’Neill, 2020). The levels of GLUT1 expression seem to correlate with the PASI score, promoting histological alterations, such as epidermal thickness, inflammatory infiltrate density, microvessel density, and Ki-67 expression (Nestle et al., 2009). Genetic or chemical targeting of GLUT1 in keratinocytes could decrease (imiquimod (IMQ)-induced) psoriasiform hyperplasia in animal models (Zhang et al., 2018; Cibrian et al., 2020a). In addition, (topical) pharmacological inhibition of GLUT1 could block inflammatory gene expression and significantly suppress inflammatory infiltration and cytokine secretion in skin biopsies from psoriasis patients, suggesting that infiltrating lymphocytes can also be targeted (Zhang et al., 2018; Cibrian et al., 2020a). Consistent with previous studies, this finding indicates that activated T cells rely on GLUT1 to expand and survive upon inflammation, as described in the following paragraphs (Macintyre et al., 2014). GLUT1 might thus serve as an attractive therapeutic target in hyperproliferative skin diseases, including psoriasis.

Given that T cells lead the way over other immunocompetent cell types in immune-related diseases, recent research has increasingly focused on T cell immunometabolism in the setting of inflammatory skin. T cells participate at various stages during psoriasis progression depending on the specific cell type, from naïve T cells to the activated helper T cells (Th1 and Th-17), the regulatory T cells, the cytotoxic and the tissue-resident memory T cells. Each cell type has a unique role, having distinct energy, differentiation, and gene expression requirements. The microenvironment regulates the above through complex metabolic networks. T cell activation, as in psoriasis, induces a drastic metabolic reprogramming necessary to cope with massive proliferation and effector subset differentiation. In this regard, glycolytic activity seems to be crucial for T cells not only as an energy source but also as a metabolic basis for anabolic biosynthesis. A detailed description of the metabolic reprogramming in T cells participating in skin inflammation is presented by von Meyenn et al. (2019). Briefly, effector T cells, including CD4^+^ and CD8^+^ T cells, largely depend on glycolytic pathways to meet their increased demands in energy and intermediate substrate requirements. Upon antigen stimulation, GLUT1 transporters are rapidly presented in the cell surface of the previously naïve T cells to facilitate glucose uptake, which is the primary source for cell survival, proliferation and cytokine production. Activated T cells undergo aerobic glycolysis, mediated by the conversion of pyruvate to lactate in the presence of oxygen. As a result, cells generate significant amounts of intermediate metabolites used for cell proliferation and ATP molecules for cell survival and function through downstream metabolic cascades (Almeida et al., 2016). Modulation of glycolysis is being exploited as a T cell immunometabolic target to regulate the inflammatory response in psoriasis mediated by effector T cells and T-helper cells. T cell glycolysis was reported to be upregulated in experimental models of autoimmune diseases, whereas its inhibition has ameliorated disease outcomes in experimental autoimmune disease models (Gerriets et al., 2015; Yin et al., 2015).

Different from naïve or activated T cells, memory T cells’ survival and function depend on oxidative phosphorylation (OXPHOS) due to the skin microenvironment characterized by reduced oxygen, glucose, and nutrient levels and increased levels of lipids. Thus, memory T cells, responsible for peripheral tissue surveillance, upregulate OXPHOS and fatty acids oxidation to produce energy for cell survival and *de novo* fatty acids and cholesterol synthesis for cell proliferation. Specifically for the skin tissue-resident memory T cells (skin TRM), energy is primarily produced through the catabolism of exogenous free fatty acids from cutaneous lipids. Furthermore, it has been proposed that the dependence of TRM cells’ survival on lipids increases with time, presenting a novel therapeutic target in disease progression and relapsing. Besides GLUT1, increased expression of the amino acid transporters of the L-type amino acid transporter ((LAT) family has been found in the epidermis of patients with psoriasis. LAT1, the primary transporter of L-Leucine, is over-expressed in activated keratinocytes and immune cells, such as T and B cells, IL-17-secreting γδ T cells, macrophages, and natural killer cells. Specific deficiency of LAT1 in activated lymphocytes seems to prevent the differentiation to Th1, Th2, and Th17 cell lineages mediated by activating the mechanistic target of rapamycin (mTOR). Also, LAT1 inhibition or deletion can decrease IL-23/1β-induced proliferation and secretion of IL-17/22 in γδ T cells, conferring protection against psoriasiform hyperplasia (Cibrian et al., 2020b).

LAT-2 and LAT-3 transporters, responsible for transporting several other amino acids, support LAT-1 function by forming heterodimers with CD-98. Inhibition of the LAT-1 transporter in the IMQ animal model did not suppress epidermal proliferation indicating the compensatory role for other transporters of the LAT family (Cibrian et al., 2020b).

Cationic Amino Acid Transporter-1and -2 (CAT1 and CAT2), the main transporters of L-Arginine, are continuously expressed by keratinocytes, promoting the metabolism of L-arginine to nitric oxide (NO). NO is an essential physiological modulator of wound repair through its effect on cell proliferation, angiogenesis and inflammation, and its production is regulated by inducible NO synthase (iNOS) and arginase (Luo and Chen, 2005). Levels of L-arginine have decreased in the blood of patients with psoriasis, which might be associated with the increased levels of arginase in psoriatic lesions, indicating a role for CAT1 and CAT2 in psoriasis (Cibrian et al., 2020a).

Sodium neutral amino acid transporter 1 (SNAT1) and alanine serine cysteine transporter ASCT2 transporters are responsible for the uptake of L-glutamine, L-alanine and L-serine, which can modulate cell differentiation and proliferation of T cells and cytokine secretion. The exact role of these transporters in inflammatory skin diseases, including psoriasis, remains to be elucidated. However, there is evidence that circulating amino acids levels are valuable markers for disease severity and response to treatment monitoring (Kamleh et al., 2015; Ottas et al., 2017).

#### 3.1.2 Molecular mechanisms of psoriasis immunοmetabolism

Metabolic and nutrient sensing pathways can coordinate immune cells’ and keratinocytes’ activation and differentiation in psoriatic skin (Cibrian et al., 2020a). The phosphoinositide 3-kinase (PI3K)/Akt/mTOR cascade acts as a critical mediator of inflammation and master sensor of the metabolic status at the intracellular level (Zhang and Zhang, 2019; Pålsson-McDermott and O’Neill, 2020). MTOR, the master sensor of nutrients and growth factors, plays a central role between the extracellular nutrient status triggers and the promotion of cell differentiation and proliferation. It is a serine/threonine kinase forming the mTORC1 and mTORC2, which regulates the anabolic and catabolic cellular processes depending on the metabolic needs and available “food.” Briefly, under starvation conditions, mTORC1 is inhibited, thus downregulating cell growth mechanisms, including protein synthesis and synthesis of lipids and nucleotides and glycolysis, while mechanisms of survival and maintenance are upregulated, including autophagy and protein degradation (UPS). On the other hand, mTORC1 downregulates cell proliferation and growth and migration (Cibrian et al., 2020a; Pålsson-McDermott and O’Neill, 2020). Several lines of evidence suggest that activated mTOR signaling seems to be involved in the pathogenesis of psoriasis through the upregulation of cell proliferation and secretion of inflammatory mediators (Patel et al., 2018; Cibrian et al., 2020a). Specifically, stimulation of mTOR is essential for T helper effector cell lineages by promoting the differentiation towards Th1, Th2, and Th17. Increased mTOR signaling has been found in proliferating γδ T cells secreting IL-17 in response to innate stimuli such as IL-23 and IL-1β. The mTOR pathway is also activated in psoriasis patients’ Treg and peripheral blood mononuclear cells. In addition, upregulation of mTOR signaling in psoriatic skin may also play a role in T cells proliferation and function, defective differentiation of keratinocytes, and their secretion of pro-inflammatory mediators (Buerger et al., 2013; Bürger et al., 2017; Cibrian et al., 2020a). Usually, mTORC1 is activated in cells of the basal layer of healthy skin, promoting their differentiation and reducing their proliferative capacity, but it is deactivated in the keratinocytes of the epidermis. Continuous activation of mTORC1 in keratinocytes has been proposed as a critical mechanism of psoriasis development. Specifically, it was shown that exposure of keratinocytes to IL-1β, IL-17A, IL-22 and TNF-α activates mTORC1 and the downstream targets, thus leading to proliferation increase and differentiation stall (Mitra et al., 2012; Buerger et al., 2017). The involvement of the Akt/mTOR pathway in the development of psoriatic plaques was further demonstrated by the increased secretion of IL-6, CXCL8, or VEGF by keratinocytes exposed to TNF, mediated by mTORC (Patel et al., 2018).

These mechanisms could explain the studies demonstrating a link between metabolic complications such as IR and obesity with psoriasis, high prevalence rates of cardiometabolic comorbidities in psoriasis, and improvement of psoriasis skin lesions after dietary intervention (Ip and Kirchhof, 2017; Mahil et al., 2019). The molecular pathways underlying the causal relationship between inflammatory skin diseases, such as psoriasis, and insulin resistance include the presence of the insulin receptors and insulin growth factors receptors (IGFR) on keratinocytes and fibroblasts and their regulation by insulin levels. Evidence from patients with psoriasis demonstrates upregulation of the PI3K/Akt pathway in peripheral blood cells and reduced levels of the Akt downstream mediator, FOXO, in psoriatic lesions (Ochaion et al., 2009; Liu et al., 2011). According to the proposed mechanism, under IR conditions, the resulting hyperinsulinemia facilitates insulin binding to its receptor IRS and IGFR despite their low affinity promoting keratinocyte and fibroblast proliferation. Moreover, overexpression of the PI3K/Akt pathway leads to the translocation of the phosphorylated FOXO to the cytoplasm, thereby preventing it from exerting its role as a cell cycle regulator (Buerger, 2018). In addition, beyond its role in T cell proliferation and function, mTOR is upregulated in keratinocytes in response to the pro-inflammatory cytokines and growth factors, promoting cell proliferation and defective differentiation (Zhang and Zhang, 2019).

Overall, our improved understanding of skin and T cell immunometabolism, coupled with the interplay between metabolic and inflammatory pathways, has led to significant progress in biomarker discovery, paving the way for novel targeted treatments, which will be discussed in the following sections.

## 4 Metabolic biomarkers in psoriasis

Studies focusing on the metabolism of psoriasis have yielded insightful findings prompting researchers to investigate potential biomarkers that will deepen our knowledge of the complex relationship between metabolism and immune responses and serve as prognostic tools.

The field focused on the study of metabolism is metabolomics, which as part of the other -omics sciences such as genomics and proteomics, has multiple applications in basic research and biomarker discovery.

Metabolomics is the gold standard for the diagnosis of inborn metabolic errors. With the emergence of sensitive equipment and methodologies, subtle changes in metabolism can be detected, as those happening during inflammatory diseases. The unprecedented advantage of analyzing the metabolome is that metabolites are the end products of cellular processes in response to genetic and non-genetic factors. Specifically, metabolites can give the researcher or clinician information on the traits that have led to a specific phenotype of an individual or a group of people with the same phenotype (e.g., disease) shaped by the combination of genetic expression, environment, epigenetic modifications and all other kinds of influences. In fact, by analyzing metabolites, one can look directly at the phenotype and see how it is affected by a disease, drug, diet and other intermediate factors (e.g., microbiota). Therefore, metabolites open a new window in discovering predictive biomarkers due to their ability to “sense” metabolic abnormalities before they lead to severe phenotype and disease symptoms. In addition, metabolomics is already employed in novel therapeutic strategies for psoriasis, aiming to shape immunometabolism and regulate uncontrolled immune responses (Sarandi et al., 2021).

We conducted a literature search for articles focused on metabolomics and psoriasis on MEDLINE PubMed. The detailed search strategy included the following keywords: (psoriasis) AND (metabolomics). We scrutinized journal articles, excluding review articles, book chapters, preprints, and articles not written in English or without access to the full article. The search was not restricted in terms of metabolomics methodology used and sample type. Obtained results were scanned for metabolic biomarkers in human psoriasis samples categorized into the following groups 1) Glycolysis, 2) TCA cycle, 3) Aminoacid metabolism, 4) Lipids metabolism, 5) Microbiome. Metabolites categorized in other groups were not included. The categories were selected based on the key metabolic derangements in keratinocytes and T cells that dominate psoriasis progression. These categories were also used to group the metabolism-based therapeutic targets of psoriasis in an attempt to obtain a better overview of the potential clinical application of psoriasis metabolic biomarkers. The following sections will discuss up-to-date findings on the metabolic biomarkers of key disturbed metabolic pathways in keratinocytes and T cell subpopulations (Table 1).

**TABLE 1:** Metabolic biomarkers in psoriasis related to energy metabolism, inflammation and insulin resistance and the gut microbiome. FA, Fatty acids; SFA, Saturated fatty acids; UFA, Unsaturated fatty acids; BCAA, Branched-chain amino acids.

Metabolic pathway	Metabolic biomarker	Type of sample	Group	Interpretation	Potential clinical application	References
Energy metabolism	Reduced glucose, lactic acid, myoinositol, and increased choline	Psoriatic lesions	10 patients with psoriasis, lesions vs. uninvloved skin and corticosteroids treated vs. untreated/100 psoriatic patients vs. 100 healthy individuals	Glucose overconsumption and changes in related metabolites in the epidermis cover energy demands	Diagnostic, response to treatment/Diagnostic, disease severity	Sitter et al. (2013), Dutkiewicz et al. (2016)
	Increased lactic acid, 2-ketoglutaric acid, aspartic acid, glutamic acid and succinic acid	Peripheral blood	10 patients with psoriasis, 10 patients with psoriasis and psoriatic arthritis and 10 control/14 psoriasis patients and 15 healthy controls	Increased amino acid mobilization from the periphery to cover energy demands in the skin	Diagnostic / Diagnostic	Armstrong et al. (2014), Dutkiewicz et al. (2016), Kang et al. (2017)
	Lower citric acid, alanine, methyl succinic acid and succinic acid	Urine	1,210 patients and 100 controls (two patient subgroups were recruited representing extreme disease activity)	Perturbed TCA cycle and alanine glucose cycle	Diagnosis and monitoring of disease activity	Julià et al. (2016)
Inflammation and Insulin resistance	Decreased arachidonic acid (Increased arachidonic-derived metabolites in lesions)	Peripheral blood	12 patients with psoriasis vulgaris and 12 healthy volunteers/epidermis specimens from 8 psoriasis patients (involved vs. uninvolved epidermis)	Pro-inflammatory state, insulin resistance	Diagnostic / Diagnostic	Hammarström et al. (1975), Li et al. (2019)
	Increased dihomo-gamma-linolenic acid	Peripheral blood	Patients with ichthyosis vulgaris, acne vulgaris or psoriasis and healthy individuals		Diagnostic	GRATTAN et al. (1990)
	Decreased total omega-3 FA	Peripheral blood	85 patients with exacerbated plaque psoriasis and 32 healthy controls	Abnormal FAs profile associated with psoriasis severity	Disease severity	Myśliwiec et al. (2017)
	Increased SFA/UFA	Peripheral blood	85 patients with exacerbated plaque psoriasis and 32 healthy controls		Disease severity	Myśliwiec et al. (2017)
	Increased BCAA	Peripheral blood	14 patients with psoriasis and 15 healthy controls	MTOR-mediated insulin resistance, inflammation and oxidation in psoriasis	Early diagnosis	Kang et al. (2017)
Gut microbiome	Increased phenol, p-cresol,3-hydroxyisovalerate	Peripheral blood	50 healthy female volunteers (administered a prebiotic beverage to 19 healthy female volunteers)	Association with keratinization and epidermal differentiation	Diagnostic	Dawson et al. (2011), Miyazaki et al. (2014), Souto-Carneiro et al. (2020), Chen et al. (2021a), Yu et al. (2021)

### 4.1 Glucose metabolism and TCA cycle

As described earlier, keratinocytes rely primarily on glucose breakdown to meet their energy demands, while local T cells employ glycolysis and OXPHOS for ATP production. Previous metabolomics studies have demonstrated reduced glucose and glycolysis-related downstream metabolites (lactic acid and myoinositol) in psoriatic lesions/skin, reflecting the metabolic changes and increased glucose uptake to cover the higher energy requirements. Notably, topical corticosteroid treatment restored glucose and myoinositol levels similar to the uninvolved skin site. Choline, responsible for the mobilization of lipids to produce energy, has also been found to increase in psoriatic lesions and to decrease in treated lesions (Sitter et al., 2013; Dutkiewicz et al., 2016). On the contrary, circulating lactate levels were higher in the serum of patients with psoriasis, together with metabolites from amino acid metabolism, including 2-ketoglutaric acid, aspartic acid, and glutamic acid (Lian et al., 2020). It has been suggested that these observations may indicate an increased mobilization of amino acids through the periphery to the inflammation site for the proliferative requirements of the skin and immune cells (Armstrong et al., 2014; Kang et al., 2017). Furthermore, metabolomic profiling of psoriasis has demonstrated a difference in the levels of metabolites participating in the TCA cycle, including citrate, methyl succinic acid and succinic acid and several essential amino acids for energy production and the generation of intermediate metabolites (Julià et al., 2016).

2-Ketoglutaric acid has been found to be higher in the serum of patients with psoriasis compared to healthy individuals but lower compared to psoriatic arthritis patients (Armstrong et al., 2014). 2-Ketoglutaric acid has a dual role as a TCA cycle intermediate and as a precursor of glutamate, which is further metabolized to proline for collagen synthesis. According to the researchers, increasing 2- ketoglutaric acid may be due to an increased need for glutamine, insulin resistance, and excess need for collagen synthesis. However, given the central role of this metabolite in multiple pathways, a causal relationship has not been established requiring additional markers.

### 4.2 Lipid metabolism

Skin is a lipid-rich and nutrient-scarce microenvironment that appears to be shaping the metabolic functions of T cells that migrate from the nutrient-rich lymphoid tissues. Even though we lack quantification studies on the differential levels of glucose, lipids and other nutrients in the skin, there is growing evidence that migrating T cells undergo metabolic adaptation within the skin. Specifically, it has been shown that skin TRM free fatty acids uptake is higher than that of peripheral T cells, either memory or effector T cells, upregulating uptake and beta-oxidation mechanisms to meet their energy and substrate demands (Pan et al., 2017).

Peroxisome proliferator-activated receptor gamma (PPAR-γ or PPARG) is a nuclear receptor responsible for lipid uptake and storage. Studies have suggested that PPAR-γ exerts a central survival mechanism for TRM metabolic adaptation in the lipid-rich environment through the upregulation of the fatty-acid-binding protein 4/5 facilitating the transport of fatty acids (Pan et al., 2017). Interestingly, the abundance of several metabolites, including unsaturated fatty acids, regulate the PPAR-γ activity and has been associated with the suppression of keratinocytes growth, suggesting that a non-pharmacological regulation of lipids would serve as a metabolic target for skin diseases (Garner et al., 1995; Ramot et al., 2015).

Metabolism of free fatty acids is mediated by carnitine, an amine that functions as a transporter of long-chain fatty acids to mitochondria for beta-oxidation. Carnitine also acts as a scavenger contributing to the clearance of amino acid metabolism intermediates. The binding of fatty acids to carnitine is mediated by carnitine palmitoyl transferase-1 (CPT-1). CPT-1 pharmacological suppression resulted in reduced production and survival of the skin TRM (Pan et al., 2017).

Metabolic profiling of fatty acids in human biofluids has yielded encouraging results regarding biomarker discovery for psoriasis and its association with disease severity and type, which has been recently reviewed elsewhere (Koussiouris et al., 2021; Sarandi et al., 2021). Overall, altered lipid metabolism in psoriasis has been associated with increased beta-oxidation, lipid synthesis, and downregulation of lipolysis (Lian et al., 2020). Many identified markers are associated with IR and inflammation, highlighting the interconnection between psoriasis and metabolic disorders. Specifically, arachidonic acid and dihomo-gamma linolenic acid levels, which have been found altered in several studies in psoriasis, are gaining increasing attention as they reflect IR and might have a predictive value for metabolic complications related to psoriasis (Hammarström et al., 1975; GRATTAN et al., 1990; Li et al., 2019; Tsoukalas et al., 2019). Of note, dysregulated patterns of arachidonic acid metabolism have been reported in several studies analyzing psoriatic lesions indicating a central role in the progression of topical inflammation and disease (Koussiouris et al., 2021). On the other hand, circulating anti-inflammatory omega-3 fatty acids were found to be lower in patients with higher PASI score, and the ratio of saturated fatty acids to unsaturated fatty acids (SFA/UFA) was positively associated with disease duration (Myśliwiec et al., 2017). Longitudinal studies of healthy populations with present metabolic dysfunctions, such as IR but no disease, will validate the predictive value of such biomarkers in psoriasis and reinforce the role of metabolic reprogramming in psoriasis.

### 4.3 Amino acid metabolism

Psoriatic lesions are characterized by extensive acanthosis of the epidermis through the hyper-proliferation of the keratinocytes. It has been suggested that changes in amino acid levels in the skin or biofluid samples of patients with psoriasis may reflect the increased demands on amino acids for collagen synthesis, cell proliferation and nucleic acids synthesis. Amino acid availability changes in the skin microenvironment can thus be a limiting factor for skin and immune cells through the mTOR pathway. In addition, branch chain amino acids (BCAA) (valine, leucine, isoleucine) and their alpha-ketoacids derivatives are emerging as potent biomarkers in psoriasis and would serve as early indicators of metabolic derangements for disease onset (Kang et al., 2017; Li et al., 2019; Zhao et al., 2019; Souto-Carneiro et al., 2020; Chen et al., 2021a). BCAA’s association with psoriasis mainly lies in their involvement as activators of the mTOR pathway promoting IR, oxidation and inflammation. In addition, BCAA’s increased levels may result in IR and metabolic disturbance through the continuous supply of the TCA cycle, causing mitochondrial overload, defective oxidation and insulin sensitivity reduction (Tsoukalas et al., 2021).

Other studies on circulating levels of amino acids have identified downstream metabolites of the glutamine/glutamate pathway, the urea cycle, and the metabolism of tryptophan and phenylalanine, which, according to researchers, are implicated in the development of psoriatic lesions (Koussiouris et al., 2021).

### 4.4 Gut microbiome

The gut-skin axis has been at the center of interest for many skin diseases, including psoriasis. The human gut is colonized by bacteria and other microorganisms, which are indispensable for the host’s homeostasis and health. Alterations in gut diversity and abundance have been linked to pathological conditions due to their central role in intestinal barrier maintenance, regulation of the immune response and protection from pathogens, production of essential nutrients such as biotin, and metabolites (short-chain fatty acids) through food digestion that fuels important metabolic pathways. Although the causal relationship between gut homeostasis disruption, known as dysbiosis, with psoriasis, is not fully established, intestine barrier permeability and epidermal differentiation seem to be key factors. The hypothesis described by Chen et al. (2020) suggests that gut dysbiosis due to diet, medication, and medical and family history leads to impaired integrity of the intestine barrier and abnormal production of microbial metabolites. Phenol, p-cresol and hippurate are endogenously produced by pathogenic bacteria and have been used as functional biomarkers of pathogens’ overgrowth (Chen et al., 2021b). Exposure of antigens and metabolites from the gut to the circulation activates the immune response mediated by Th-17 cells, a dominant population in psoriasis, and suppression of the Tregs. In addition, phenol and p-cresol have been associated with impaired keratinization and disrupted epidermal differentiation (Dawson et al., 2011; Miyazaki et al., 2014). Conversely, 3-hydroxyisovalerate is a marker indicating the abundance of biotin-dependent bacteria, including certain *Lactobacillus* species that are beneficial to the host. In a previous human study, 3-hydroxyisovalerate was found to be significantly altered in patients with generalized pustular psoriasis (Yu et al., 2021). Overall, metabolomic studies investigating microbial metabolites in psoriasis are very scarce, and most studies use DNA-based microbiome analysis. However, neither DNA technique can capture the microbiome’s metabolic function, which can be directly linked to its targets (metabolism, immune response, etc.). Even though metagenomics has emerged as more sensitive to detecting the diversity of present microbiomes, according to recent study reviews, microbiome composition and not abundance are strongly associated with psoriasis, and future better-designed studies will validate existing findings (Sikora et al., 2020). Qualitative and quantitative analysis of the metabolites related to gut dysbiosis would open a new path in biomarker discovery of psoriasis through the involvement of gut microbiota and novel metabolic therapeutic targets.

## 5 Metabolic targets for psoriasis treatment

Current treatment approaches for psoriasis are mainly based on disease severity and the presence of psoriatic arthritis. In mild to moderate cases, treatment mainly includes topical anti-psoriatic agents, i.e., topical steroids, topical calcineurin inhibitors, vitamin D analogs, and retinoids), while, in severe cases, systemic, classical or biological therapies are usually required (Armstrong and Read, 2020).

Several therapies targeting immunometabolism exist, including pharmacological agents with known anti-inflammatory properties that target metabolism, such as metformin, dimethyl fumarate and methotrexate, reviewed by Pålsson-McDermott and O’Neill (2020). However, novel targeted therapies are emerging based on immune cells’ and keratinocytes’ different metabolic demands. The distinctive advantage of these therapeutic strategies lies in the selectivity of the drug against cells undergoing metabolic reprogramming, which leaves normal cells unaffected and preserves tissue homeostasis.

### 5.1 Glycolysis

A promising metabolic target of psoriasis is the dependence of activated keratinocytes and T cells on glucose and the upregulation of the Glut1 transporter. Pharmacological inhibition of Glut1 with the agent WZB117 resulted in the suppression of the inflammatory response in skin biopsies of patients with psoriasis and decreased hyperplasia in animal models of psoriasis (Zhang et al., 2018). In this direction, the glycolysis inhibitor 2-deoxy-D-glucose (2-DG) has been proposed as a potential therapeutic target in Th-17-dependent diseases, including psoriasis. Specifically, treatment with 2-DG reduced the skin thickness and improved the skin lesions in a psoriatic mouse model (Huang et al., 2019; Liu et al., 2021). In this regard, a low-glycemic diet has been suggested to improve psoriasis as a supportive treatment, especially in overweight patients (Ford et al., 2018). It has been previously shown that a very low carbohydrate ketogenic diet protocol improves the expansion of psoriatic lesions with the concurrent improvement of biochemical and inflammation markers in patients with psoriasis. The nutritional intervention resulted in the reprogramming of several metabolic pathways, to the levels of healthy control, with the most significant being dysmetabolism and amino acid metabolism possibly linked to keratinocytes hyperproliferation (Castaldo et al., 2021).

### 5.2 Amino acid metabolism

Additional metabolic targets of psoriasis include amino acids metabolic pathways involved in Th17 cell differentiation and activation, which are indispensable for disease progression. CB-839, a glutaminase inhibitor, was reported to inhibit Th17 proliferation and promote Th1 expansion without altering Tregs survival leading to Treg skewing phenotype. Glutaminase inhibition and subsequent deprivation of glutamate and aspartate promoted glycolysis and the contribution of glucose-derived production of amino acids, possibly contributing to the excess production of ROS (Johnson et al., 2018). Similarly, treatment with halofuginone, a plant-derived inhibitor of glutamyl-prolyl-tRNA synthetase, which is responsible for the attachment of glutamic acid to tRNA during protein synthesis, leads to amino acid starvation response. As a result, TH17 differentiation is ablated, which is a protective mechanism in mice with Th-17-driven experimental autoimmune disease (Sundrud et al., 2009). In addition to amino acid metabolism reprogramming, rapamycin-mediated inhibition of mTORC1 defines T-cells’ differentiation and polarization, promoting a Treg-enriched phenotype and downregulation of Th17 expansion. Treatment with rapamycin has been reported to exert beneficial effects in patients using rapamycin-based topical therapy, validated in experimental animal models of psoriasis (Buerger, 2018). mTOR inhibition through a fasting diet has been shown to exert beneficial effects on multiple levels beyond weight loss, including restoring the Treg/Th17 balance through metabolic rewiring (Ford et al., 2018; de Cabo and Mattson, 2019). Specifically, in psoriasis, a positive correlation between fasting and disease activity decrease has been observed in an observational study (Damiani et al., 2019).

### 5.3 Lipid metabolism

Moreover, the dependency of TRM cells migrating from the periphery to the lipid-rich skin on lipid metabolism has emerged as a potential anti-psoriatic strategy. Specifically, these cells upregulate the uptake and breakdown of fatty acids to adjust to the skin microenvironment. Thus, inhibition of either of these mechanisms may prove beneficial. Specifically, treatment with etomoxir, a carnitine-palmitoyltransferase one inhibitor, significantly reduced the TRM population and improved the psoriatic phenotype (Pan et al., 2017). Furthermore, dietary interventions have also been regarded as essential strategies in the metabolic reprogramming of patients with psoriasis, given the negative impact of saturated fats-rich diets on disease severity (Herbert et al., 2018; Higashi et al., 2018). In addition, several reports on the overproduction of arachidonic acid-derived eicosanoids in psoriatic skin lesions (Koussiouris et al., 2021). Hence, (dietary) supplementation with n-3 fatty acids might be a helpful adjuvant in psoriasis treatment.

### 5.4 Dysbiosis

Restoration of the intestinal microbiota as part of an anti-psoriatic treatment strategy has gained increasing attention. Probiotics, prebiotics and synbiotics (the simultaneous administration of pro- and prebiotics) are some widely used methods to restore the balanced microbial community of beneficial and harmful living organisms in the gut. Some clinical studies report a beneficial effect of probiotics/prebiotics administration on disease severity assessed by PASI score and inflammation markers, including CRP and interleukin levels (Groeger et al., 2013; Navarro-López et al., 2019; Moludi et al., 2022). Besides the enrichment of beneficial microorganisms, limiting the expansion and activity of potentially harmful organisms that support the psoriatic pro-inflammatory state is crucial. Adherence to the Mediterranean diet, one of the most extensively studied diet-related anti-inflammatory strategies, has been reported to improve the psoriatic phenotype. However, the role of the gut microbiome is not clear. In this sense, several other diets have been reported to improve patients’ quality of life with psoriasis depending on their comorbidities, such as a gluten-free diet when coeliac disease is present. Other diet-related anti-psoriatic strategies where the role of the gut microbiome has been explored include the administration of bioactive compounds such as polyphenols, omega-3 fatty acids and other natural compounds (Chung et al., 2022).

### 5.5 Vitamin D

Vitamin D is a widely used adjuvant therapy for psoriasis due to its known immunomodulatory effects but also through regulating the activity and proliferation of VDR-expressing keratinocytes. A growing number of studies showing a therapeutic effect of vitamin D treatment on psoriatic lesions and inflammatory responses were recently reviewed by Brozyna et al. However, some contradicting results stemming from differences in the study design hamper the establishment of vitamin D supplementation as part of the treatment of psoriasis. Among limitations, the most important is that the efficacy of vitamin D administration is assessed in clinical trials that are designed based on protocols that evaluate drug response, where the effect is immediate. In contrast with drugs, the impact of nutrient supplementation on disease activity requires more extended periods of treatment, the presence of other nutrients, called cofactors, and sufficient levels not only in the periphery but at a cellular level to exert its biological function (Scragg, 2018; Boucher, 2020; Tsoukalas and Sarandi, 2020). The VITAL randomized control trial showed that vitamin D and omega-3 supplementation for 5 years was associated with reduced autoimmune disease incidence, including psoriasis, with the most pronounced effect observed 2 years after the initiation of the intervention (Hahn et al., 2022). In addition, nutrient efficacy on disease activity needs to be tested in combination with immunomodulatory drug agents, especially in severe cases, and not as comparators since they target distinct yet complementary biological pathways. Beyond the beneficial effects on immune system response and keratinocyte activity, recent reports show that the administration of vitamin D restores gut microbiota balance, serving as a possible mode of action for its anti-psoriatic effects and, more importantly, the response variation observed in many patients (Singh et al., 2020). As a general remark, there is increasing evidence that the gut microbiome plays a significant role in disease progression and treatment response (Yeh et al., 2019). Therefore, restoration of the microbial community can directly or indirectly benefit patients receiving therapy, while gut microbiome biomarkers may serve as predictive markers of treatment selection.

## 6 Combinatorial treatment of psoriasis

Psoriasis is a skin disease with many comorbidities, a high risk of relapse and significant deterioration of patients’ quality of life. The impact of psoriasis on the quality of life of patients is comparable to that of cancer, cardiovascular disease and depression, while it is responsible for 5.6 million all-age disability-adjusted life-years (DALYs) according to the 2016 Global Burden of Disease Study report (Global, 2017). As a result, patient satisfaction, adverse effects and costs have increased the need for novel therapeutic targets. In addition, treatment efficacy is mainly evaluated by skin clearance. However, it has been suggested that patient-reported outcomes, such as the dermatology life quality index (DLQI), should be included in the standardized outcome measures of the intervention (Strober et al., 2019). However, this applies primarily in mild to moderate severity cases that do not receive biological therapy because in severe cases under systemic treatment and well-tolerated disease progression, the satisfaction rate of patients and physicians are high (Florek et al., 2018). Yet, in severe psoriasis cases under systemic treatment, even though lesions might be significantly reduced, the metabolic complications of psoriasis and the high risk for other chronic diseases should not be overlooked. In cases with mild psoriasis where patients do not meet their goals or the lesions require long-term use of medications, patients seek nutritional-based interventions that have minimum adverse effects and can be integrated into their daily routine (Afach et al., 2021). Although the efficacy of nutritional strategies on disease activity has not been deciphered, there is growing evidence that metabolic rewiring would be a valuable tool against psoriasis progression and restoration of the healthy metabolic state (Cibrian et al., 2020a). However, accumulating lines of evidence and studies, including the present paper, indicate the crucial role of metabolic networks, including the metabolites, the enzymes and the cofactors on the crosstalk of immune and tissue cells driving psoriasis pathogenesis. Therefore, combining drug-based treatment to fine-tune the immune response and the systemic metabolism with nutritional plans tailor-made to provide the nutrients required to counterbalance the metabolic deregulation of psoriasis would have significant benefits. Specifically, first-line treatment might include the management of excessive inflammatory-mediated symptomatology with systemic treatment (biological and/or metabolic) for the short term. Then, treatment would consist of diet, lifestyle changes and well-tolerated topical treatment to prevent disease relapse or manifestations of other systemic inflammation diseases, applicable for the long-term with minimum side-effects and economic burden for the patient and the healthcare system (Nestle et al., 2009). Table 2 presents therapeutic opportunities for psoriasis targeting metabolic pathways through pharmacological agents and supportive dietary recommendations based on available experimental data. Available study results on the efficacy of metabolic targeting of psoriasis can be grouped into four categories; Energy metabolism, Aminoacids metabolism, Lipids metabolism, and Gut microbiome. Targeting specific metabolic networks of affected cells in psoriasis to reprogram pro-inflammatory cells to their normal state is an attractive approach to increase efficacy and reduce relapse and side effects.

**TABLE 2:** Therapeutic opportunities for psoriasis targeting metabolic pathways through pharmacological agents and supportive dietary recommendations.

Metabolic pathway	Metabolic target	Pharmacological intervention	Cellular effect	Psoriatic phenotype effect	Supportive dietary intervention	References
Glucose metabolism	GLUT	WZB117	Glucose uptake inhibition	Decreased inflammation in human skin biopsies and reduced hyperplasia in mice	Low glycemic diet	Ford et al. (2018), Zhang et al. (2018), Castaldo et al. (2021)
		2-DG	Glycolysis inhibition	Skin thickness reduction and improved skin lesions in mice	Low glycemic diet	Ford et al. (2018), Huang et al. (2019), Castaldo et al. (2021), Liu et al. (2021)
Aminoacid metabolism	Glutaminase	CB-839	Glutamine metabolism inhibition	Restoration of Treg levels and decrease of pro-inflammatory Th17 population	—	Johnson et al. (2018)
	Glutamyl-prolyl-tRNA synthetase	Halofuginone	Amino acid starvation response	Ablation of Th-17 population in Th-17 autoimmune experimental model	—	Sundrud et al. (2009)
	mTORC1	Rapamycin	Inhibition of cellular proliferation and activity	Restoration of Treg levels and decrease of pro-inflammatory Th17 population having beneficial effects in mice	Fasting diet	Buerger (2018), Ford et al. (2018), de Cabo and Mattson (2019)
Lipid metabolism	Carnitine-palmitoyltransferase 1	Etomoxir	Inhibition of fatty acids oxidation	Reduction of TRM population with subsequent improvement of psoriatic phenotype	Low saturated and high omega-3 polyunsaturated fatty acids diet	Pan et al. (2017), Herbert et al. (2018), Higashi et al. (2018)
Gut Microbiome	Beneficial gut microbiota	Probiotics/Prebiotics	Restoration of the balance between beneficial and harmful microorganisms in the gut	Improved disease severity and inflammation markers decrease	Mediterranean Diet/gluten-free diet (if coeliac disease coexists)	Groeger et al. (2013), Navarro-López et al. (2019), Moludi et al. (2022)

## 7 Discussion and concluding remarks

Psoriasis is an inflammatory disorder affecting mainly the skin. However, new evidence shows that multiple associated complications in distant tissues can develop into comorbidities such as CVD and metabolic syndrome. Psoriatic lesions are characterized by hyperplasia of the epidermis due to the hyperproliferation of keratinocytes and delayed apoptosis, originating from the additive effects of environmental and genetic factors and mediated by complex crosstalk between immune and skin cells. Distinct changes in the metabolism of immune cells, mainly activated T cells, tissue-residing memory cells, and keratinocytes, have been linked to the critical pathogenetic mechanisms of psoriasis, including activation, increased proliferation and differentiation of the involved cells. Dysfunctional apoptosis of keratinocytes plays a crucial role in epidermal hyperplasia, possibly mediated by abnormal cytokine secretion by the affected T cells (Wang et al., 2020). Anti-psoriatic treatments that target keratinocytes, such as UV radiation or vitamin D3, have been shown to increase apoptosis rates in the lesional epidermis providing the basis for further research in therapy (Fukuya et al., 2002). In terms of metabolic regulation of apoptotic pathways, lipids have been demonstrated to act as pro-apoptotic or anti-apoptotic agents in a cell-specific manner, possibly related to the beneficial effect of essential fatty acids supplementation to keratinocyte growth (Garner et al., 1995; Wójcik et al., 2020).

Our understanding of the intertwined processes of metabolism, cell function and immune responses in psoriasis is still growing. However, metabolic disruptions in energy production pathways, amino acids and lipids metabolism and dysbiosis seem to be common, prompting us to look for novel diagnostic and therapeutic tools. Although validation studies are required, up-to-date studies have identified several metabolic biomarkers associated with the distinct metabolic fingerprint of psoriasis or associated metabolic complications such as IR. These metabolic pathways can also be targeted through drug inhibitors to limit the immune response and keratinocyte hyperproliferation, stalling disease progression. In addition, tailor-made nutritional plans to restore metabolic imbalances, thus protecting unaffected cells and promoting anti-inflammatory and resolution pathways, can contribute to the long-term control of psoriasis. However, metabolomic studies have several limitations, including the variability between studies due to the lack of a standardized method and study design that hampers reproducibility and interpretation of the findings. In addition, small sample size, different population characteristics and lack of validation datasets significantly stall the biomarker discovery process. The metabolic rewiring of cells that promote inflammation and psoriasis progression to their normal state to resolve inflammation is an exciting yet growing field, and future clinical trials are needed to investigate their application in human real life settings.

To conclude, monitoring metabolic fluctuations as part of the early diagnosis of metabolic comorbidities, prevention and treatment optimization has emerged as a powerful strategy for psoriasis and ongoing ventures report encouraging findings. With the use of metabolic biomarkers to close monitor the response to treatment and overall metabolic health, clinicians will be able to provide long-term therapy for psoriasis and prevention, early diagnosis and treatment of related comorbidities. Future studies in this direction should focus on longitudinal interventional studies to validate these biomarkers and large metabolomics studies to report differences across populations.
